# Successful Laparoscopic Management of Pericaecal Hernia Causing Small Bowel Obstruction

**DOI:** 10.7759/cureus.34663

**Published:** 2023-02-06

**Authors:** Hans Mare, William Tjhin

**Affiliations:** 1 General Surgery, Rockingham General Hospital, Rockingham, AUS

**Keywords:** adhesiolysis, closed-loop obstruction, pericecal internal hernia, surgical or open or laparoscopic and adhesive small bowel obstruction or intestinal obstruction, small-bowel obstruction

## Abstract

Management of small bowel obstruction varies depending on the cause and clinical status of patients. While most cases can be managed conservatively, a not-insignificant proportion of patients undergo surgical intervention. Laparotomy has long been the default approach for entering the abdomen in cases requiring surgical intervention, with laparoscopy largely being avoided due to abdominal distension and the risk of perforating bowels on entry.

We present here the case of a 54-year-old woman who presented with signs and symptoms as well as radiological evidence of a closed-loop small bowel obstruction in her right lower quadrant. Following a brief period of nasogastric decompression, her abdominal distension improved, allowing for a laparoscopic entry where a pericaecal hernia was noted to be the cause of her obstruction. Extensive adhesiolysis without the use of an energy device successfully allowed for the reduction of the bowel contained within. No bowel resection was performed and the patient was discharged home on day 3 following her procedure.

This case report successfully demonstrates the utility of using laparoscopy as an alternative to laparotomy in patients with a small bowel obstruction secondary to an internal hernia.

## Introduction

Internal hernia is an important consideration as a differential diagnosis in patients with intestinal obstruction. Defined as a protrusion of abdominal contents through a peritoneal or mesenteric orifice, internal hernias most commonly manifest as small bowel obstruction in adults [1]. Of the different types of internal hernias, pericaecal are diagnostically challenging, exceedingly rare, and usually managed through an emergency laparotomy [1,2]. Pericaecal hernias occur as a result of congenital or acquired defects in the caecal mesentery, through which bowel loops herniate [1]. We present the case of a 54-year-old female who presented with small bowel obstruction secondary to a pericaecal hernia, which was successfully managed through laparoscopy as an alternative to laparotomy.

## Case presentation

**Figure 1:**
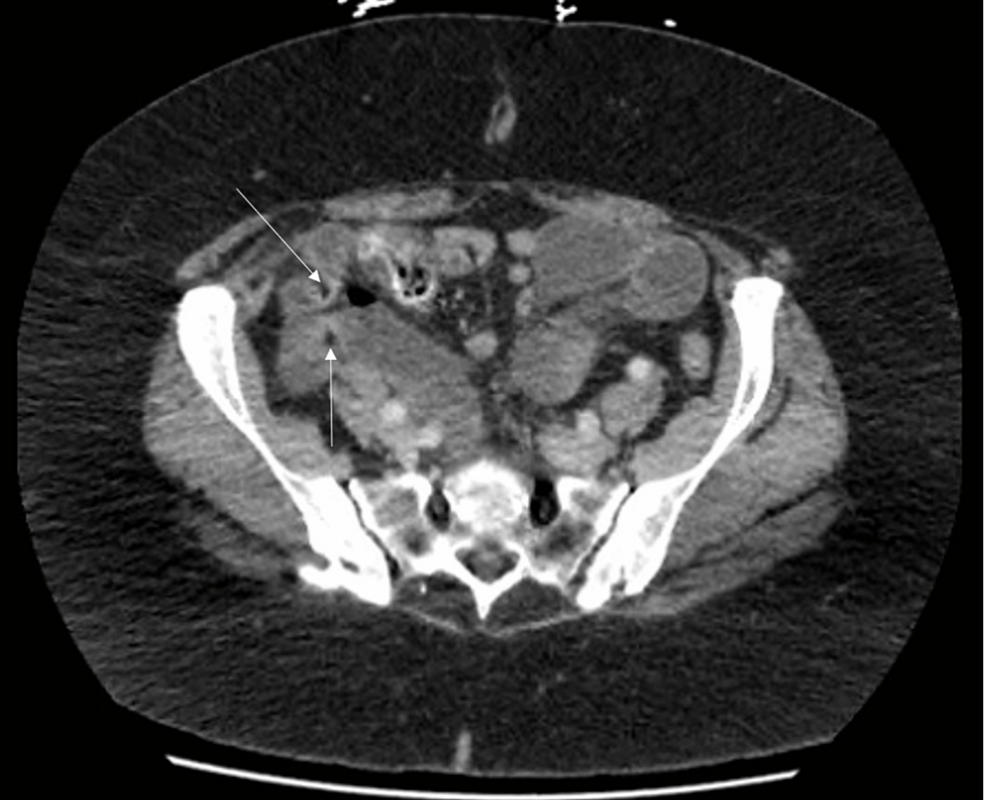
Axial view of computed tomography scan of the abdomen Arrows indicate transition points of the small bowel within pericaecal hernia.

A 54-year-old woman presented to the emergency department with a one-day history of colicky right iliac fossa pain with migration to the epigastrium. The pain was associated with vomiting and exacerbated by oral intake. The patient had previously undergone multiple laparoscopic procedures, including appendicectomy, cholecystectomy, gastric band insertion, band removal, and sleeve gastrectomy. On examination in the emergency department, the patient’s observations were within normal limits. A soft, distended abdomen was noted with tenderness in the right iliac fossa. A computed tomography (CT) scan was performed in response to a lack of relief from analgesia and demonstrated a closed-loop small-bowel obstruction with multiple transition points around a single loop in the right lower quadrant, lateral and posterior to the caecum, likely secondary to adhesions from previous appendicectomy (Figure 1). A nasogastric tube was promptly inserted in order to decompress the proximal gastrointestinal tract, therefore increasing intraperitoneal space and reducing the risk of bowel injury intraoperatively. The patient then proceeded to a diagnostic laparoscopy.

**Figure 2:**
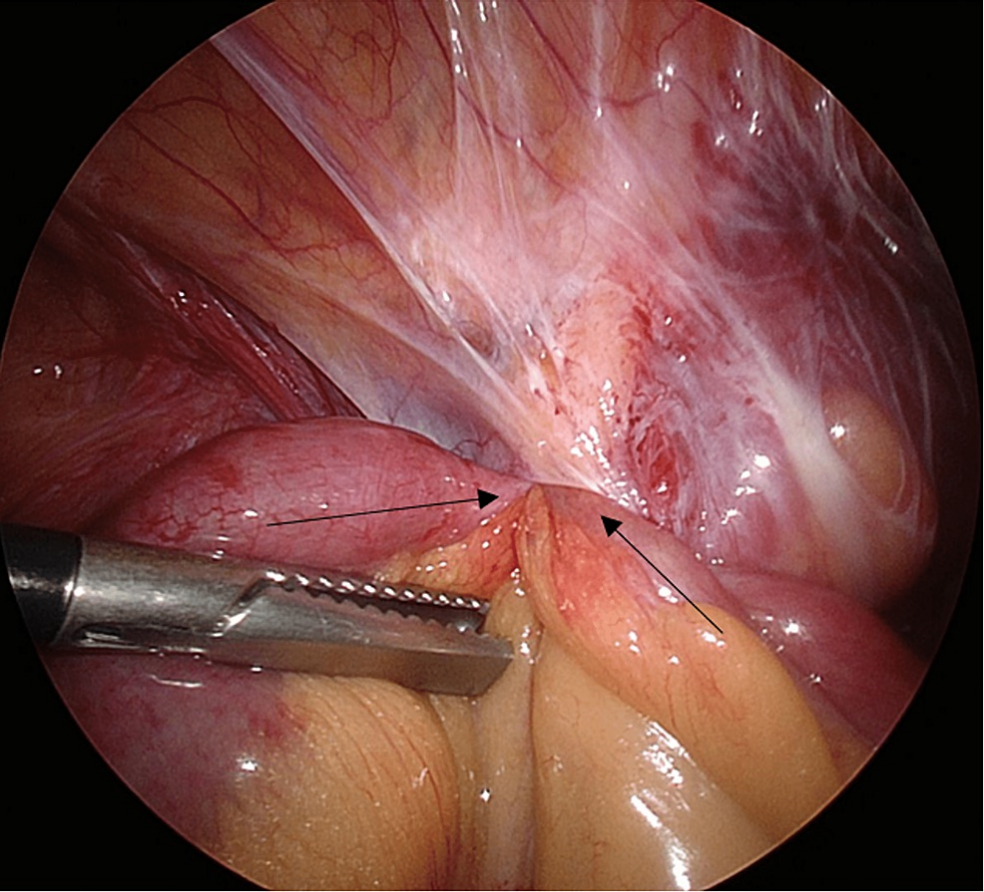
Laparoscopic view of small bowel herniating into pericaecal orifice Arrows indicate entry and exit of bowel loops in relation to pericaecal hernia.

**Figure 3:**
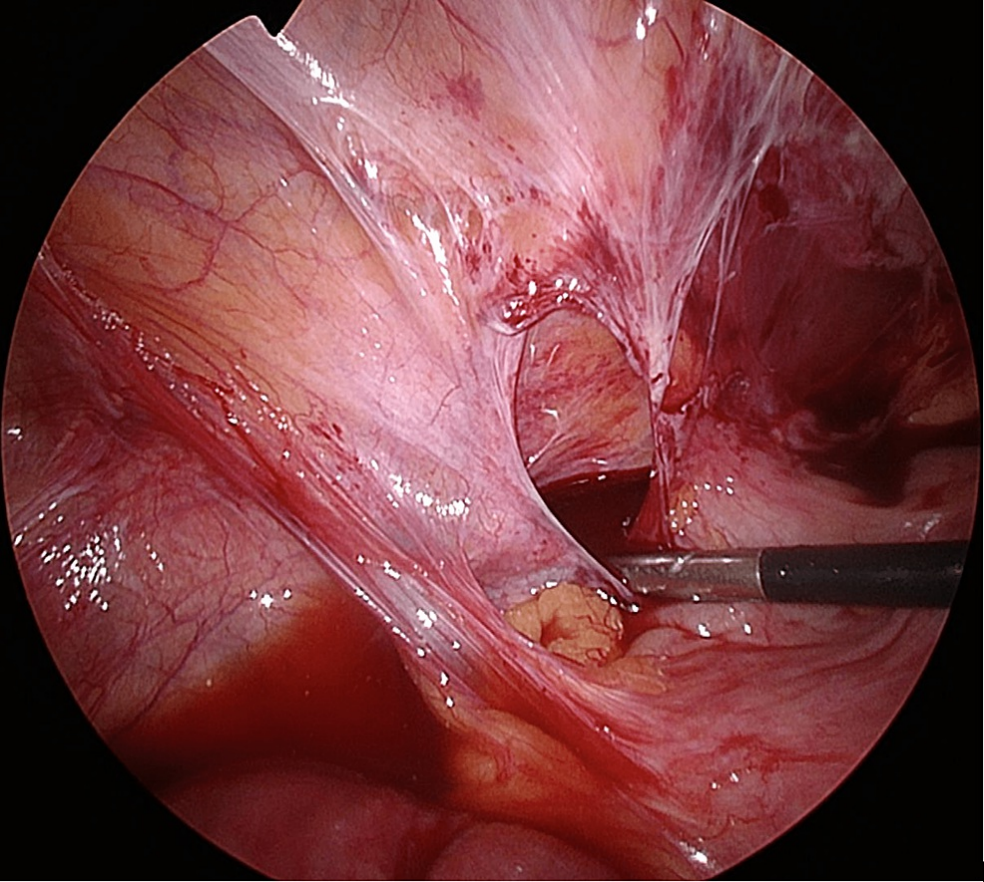
Laparoscopic view of pericaecal hernia orifice following successful reduction of small bowel within

Laparoscopic Hasson entry was achieved through the umbilicus, where CT imaging showed omental space with no bowel in the vicinity. A right colon adhesion plate to the right lateral abdominal wall with a small, tight opening around the caecum was found to have created a cocoon through which a 20-cm length of mid-small bowel herniated (Figure 2). This opening was widened, and extensive adhesiolysis was performed without the use of an energy device to liberate the contained bowel (Figure 3). An a-trac laparoscopic grasper was used on the mesentery instead of the bowel during reduction. The bowel contained within was initially dusky; however, a return of colour and vigour was observed after a pause, indicating viable bowel. The remnant pocket sac was dissected and opened to prevent a recurrence. A small bowel resection was not performed. The patient had an uncomplicated inpatient recovery and was discharged home on day 3 post-operatively following the gradual upgrade of oral dietary intake. Her follow-up outpatient review 25 days post-operatively showed normal wound healing, normal bowel function, and no incisional hernia or other complications secondary to her procedure.

## Discussion

This patient’s history and examination were suggestive of a small bowel obstruction. While her CT scan was useful in providing a diagnosis of a closed-loop bowel obstruction as well as an appropriate laparoscopic entry site, it did not detect the specific presence of a pericaecal internal hernia. Internal hernias are defined as a protrusion of a viscus, usually small bowel, through a peritoneal or mesenteric aperture within the abdominal cavity. Internal hernias are uncommon and typically manifest with a small bowel obstruction. Approximately 0.6-5.8% of small bowel obstructions in adults can be attributed to internal hernias, and pericaecal hernias are rarer still, constituting up to only 6-13% of all internal hernias [1,3]. Closed-loop small bowel obstructions that occur from internal hernias can rapidly progress from incarceration to strangulation and full-thickness bowel necrosis [4].

Pericaecal hernias emerge through an aperture that develops within the peritoneal recess formed by local folds of the peritoneum [5]. Four subtypes of pericaecal hernias have been described based on the anatomic location of the recess, including ileocolic, retrocolic, ileocaecal, and paracaecal [6]. Most commonly, the herniated bowel loop consists of an ileal segment protruding through a defect in the caecal mesentery and extending into the right paracolic gutter [3]. Patients with pericaecal hernias present in a manner similar to those with other types of internal hernias, however the location of symptoms is typically in the right lower quadrant. Pericaecal hernias can therefore be mistaken for appendiceal abnormalities [3].

Laparotomy has long been the standard operative approach to managing acute small bowel obstructions, particularly those caused by internal hernias [7]. Laparoscopy for small bowel obstruction was previously deemed inappropriate due to difficulties in establishing a working space, decreased visibility at the site of obstruction, and an increased risk of injury to the distended bowel [7]. While the feasibility of laparoscopy over laparotomy has since been proven, showing benefits such as a shorter hospital stay and fewer major complications, limited information exists for its specific use in small bowel obstructions caused by internal hernias [8]. However, there are an increasing number of case reports and studies demonstrating success in treating small bowel obstructions caused by internal hernias via laparoscopy [9]. Based on the available literature, there is no clear consensus regarding the appropriate selection of patients for laparoscopy in cases of small bowel obstruction of any cause. Studies have however suggested that the decision should be based on the surgical experience and abilities of the assessing surgeon and that a patient in shock should have no delay to theatre for a laparotomy [10,11]. In our experience, the decision to proceed with laparoscopy in cases of small bowel obstruction also depends on the success of decompression by nasogastric tube, the patient's body habitus, and the morbidity associated with laparotomy.

To the best of our knowledge, this is one of the few reported cases of a pericaecal internal hernia with small bowel obstruction being managed successfully through laparoscopy. Fewer than 40 cases of pericaecal hernias have been reported in the literature, with the vast majority being managed through laparotomy. Only eight cases have reportedly been managed laparoscopically [1,9,12].

## Conclusions

Even though it is rare, the consideration of a pericaecal hernia causing small bowel obstruction as a diagnosis is important in the patient presenting with right lower quadrant pain, particularly when signs and symptoms of obstruction are present. We believe this case successfully demonstrates the utility of using a CT scan in diagnosing small bowel obstructions and surgical planning in patients with undifferentiated abdominal pain. It also validates the practicality of laparoscopic surgery as an alternative to laparotomy in managing pericaecal hernias causing small bowel obstruction in patients where nasogastric decompression is possible. 
